# Antibiotic Prophylaxis After Urethroplasty: A Review of the Literature

**DOI:** 10.3390/jcm14113915

**Published:** 2025-06-02

**Authors:** Ellen M. Cahill, Hiren V. Patel, George E. Koch, Joshua Sterling

**Affiliations:** 1 Department of Urology, Yale University School of Medicine, New Haven, CT 06510, USA; ellen.cahill@yale.edu; 2 Department of Urology, The Ohio State University Wexner Medical Center, Columbus, OH 43210, USA; hiren.patel@osumc.edu (H.V.P.); george.koch@osumc.edu (G.E.K.); 3 Department of Urology, The University of Washington, Seattle, WA 98195, USA

**Keywords:** urethral stricture, urethroplasty, antibiotic prophylaxis, foley catheter, urethral reconstruction, radical prostatectomy, hypospadias repair, pyeloplasty

## Abstract

Urethroplasty is a highly effective surgical treatment for urethral stricture disease. While overall complication rates are low, surgical site infections and urinary tract infections (UTIs) represent the most common complications. Due to the prolonged need for catheterization following reconstruction, many urologists place patients on extended antibiotic prophylaxis postoperatively. However, antibiotic stewardship is important given the risks of antibiotic overuse including opportunistic infections and the emergence of multidrug-resistant organisms. There are currently no established guidelines for the management of antibiotics for patients undergoing urethroplasty, specifically with regard to postoperative prophylaxis through the time of foley catheter removal. In this review, we examine the current literature regarding antibiotic prophylaxis and urethroplasty. Research has shown no clear benefit for extended antibiotic prophylaxis in preventing symptomatic urinary tract infections or stricture recurrence. This is congruent with evidence from other urologic procedures requiring indwelling catheters and/or stents including radical prostatectomy, hypospadias repair, and pyeloplasty. Prospective, randomized trials are needed to further understand the impact of antibiotic prophylaxis on both urethroplasty outcomes and its broader impact on recurrent UTIs and microbial resistance.

## 1. Introduction

Urethral stricture disease affects an estimated 349 of every 100,000 men in the United States, with an annual incidence of approximately 0.9% [1]. Urethral strictures can be managed endoscopically with either dilation or urethrotomy; however, these treatments are associated with a low likelihood of cure and often require repeat procedures [2]. Urethroplasty is a definitive surgical procedure utilized to repair urethral strictures. As per the American Urological Association (AUA) guidelines, urethroplasty can be offered to all men with urethral strictures, and is specifically recommended for strictures > 2 cm, penile urethral involvement, and recurrences after endoscopic management [3]. Urethroplasty can be performed using anastamotic or substitution approaches, depending on the location and length of the stricture. In either case, a urethral catheter is placed postoperatively and maintained for 1–4 weeks to divert urine away from the anastomosis and prevent extravasation.

The most common complications after urethroplasty include wound infections and urinary tract infections (UTIs), each of which have been found to occur in 3–7% of cases [4,5,6]. Perioperative antimicrobial prophylaxis (AP) aims to reduce the risk of surgical site infections (SSIs) and UTIs. According to the AUA guideline on urethral strictures, antibiotics guided by preoperative urine cultures should be given prior to surgery to reduce SSIs [3]. However, the benefits of AP must be weighed against the potential risks, including suppression of normal flora, which can lead to *Clostridium difficile*, colonization or infection with multidrug-resistant (MDR) organisms, all of which can increase the overall cost of treating urethral strictures [7,8]. In an effort to decrease unnecessary AP, the AUA published a “Best Practice Statement on Urologic Procedures and Antimicrobial Prophylaxis”, initially in 2008 and updated in 2020. The Best Practice Statement recommends a single dose of perioperative IV cephalosporin, and, more broadly, “the least amount of antimicrobials needed to safely decrease the risk of infection to the patient should be used to minimize antimicrobial-related adverse effects and decrease the risk of drug-resistant organisms [9]”. Despite these recommendations, many urologists have continued to utilize prolonged courses of antibiotics, both treatment and prophylactic doses, across procedures in practice [10,11,12,13].

Given the need for prolonged foley catheter use after urethroplasty, patients are at risk for catheter-associated postoperative infections through the time of removal. Furthermore, both UTIs and SSIs following urethroplasty have been theorized to lead to inflammation at the anastomosis, which can impair wound healing and potentially lead to fistula formation or stricture recurrence. Regarding foley catheter removal, the AUA Best Practice Statement advises that AP “may be considered” at the time of trial of void, catheter removal, or drain removal; however, no further guidelines exist regarding choice of agent or duration [9].

Tremendous variability exists amongst urologists for AP both before and after urethroplasty. The goal of this review is to assess the current literature and future areas for investigation regarding the role of AP in this patient population.

## 2. Materials and Methods

A literature search using PubMed/MEDLINE, Embase, Cochrane library, and Scopus was carried out to perform a comprehensive structured narrative review of articles published through 1 January 2025 using the following search terms: “urethroplasty” AND “antibiotics”, “urethral reconstruction” AND “antibiotics”, “radical prostatectomy” AND “antibiotics”, “ureteral reconstruction” AND “antibiotics”, “hypospadias repair” AND “antibiotics”, “pyeloplasty” AND “antibiotics”. Selected articles were required to be original articles written in English. Systematic reviews, original articles, and case reports/series were included. Commentaries and news articles were excluded. The studies were independently reviewed by author E.C. References of papers were reviewed for potential missed studies. All articles identified in previous systematic reviews were included.

## 3. Current Recommendations and Practice Patterns

A summary of societal guidelines is shown in Table 1. Preoperatively, both the AUA Best Practice Statement and the AUA guideline on urethral strictures recommend all patients provide a urine culture and receive culture-directed preoperative antibiotics to reduce SSI [3,9]. Similarly, the European Association of Urology (EAU) recommends screening for and treating asymptomatic bacteriuria prior to urinary tract manipulation [14]. In terms of intraoperative management, the AUA guidelines recommend a single dose of a first- or second-generation cephalosporin before open surgery involving the urinary tract [3]. The EAU recommends intraoperative antibiotics as per the local antibiotic resistance profile [14,15]. Similar recommendations are provided by the Infectious Disease Society of America (IDSA) [16]. Beyond this first dose, there are no specific guidelines for perioperative or postoperative antibiotics in patients undergoing urethroplasty.

Regarding AP while a foley catheter is in place, the AUA Best Practice Statement states that AP is “not indicated for the duration of indwelling catheterization in the postoperative period for the reduction of SSI as they do not reduce the risk of a catheter-associated UTI (CAUTI) [9]”. The EAU guideline states “Although most urologists continue with postoperative antibiotics upon and even beyond catheter removal, there is no evidence that such prolonged administration reduces the infective complication rate [14]”. At the time of catheter removal, the AUA guidelines state that AP “may be considered at the time of clinical procedures such as trials of voiding, or removal of catheter or drain tubing, or stent or nephrostomy tube, especially when other patient and procedural risk factors are present”, but no further guidelines exist regarding choice of agent or duration in these specific scenarios [9]. The IDSA similarly states that AP at the time of catheter removal “may confer a benefit for prevention of symptomatic UTI for some patients” [17].

Given the lack of clear guidelines regarding postoperative AP in patients undergoing urethroplasty and concerns regarding increased risk of infection and stricture recurrence in this patient population, practice patterns vary, and many urologists prescribe antibiotics through foley catheter removal. McDonald and Buckley surveyed 34 reconstructive urologists who performed high-volume urethroplasties regarding antibiotic use for these patients. Most respondents reported ordering a preoperative urine culture and treating any positive culture with culture-directed antibiotics [12]. All respondents utilized intravenous (IV) antibiotics intraoperatively; however, the agents varied, with aminoglycosides, cephalosporins, and penicillins being the most common and the majority utilizing two agents. Most discontinued IV antibiotics within 24 h and continued oral antibiotics until catheter removal, with the most common agents being nitrofurantoin and fluoroquinolones [12]. A 2021 survey of 142 reconstructive urologists found that 72% continue oral AP until foley catheter removal [13].

## 4. Urethroplasty and Postoperative Infectious Complications

Following urethroplasty, patients typically require prolonged foley catheter placement (1–4 weeks). This patient population is certainly at risk for developing a postoperative UTI given the higher likelihood of baseline urinary stasis, prior instrumentation, and need for urinary catheters [19]. Many of these patients may be colonized preoperatively, which is further confounded by the addition of a foley catheter after surgery. The presence of a foley catheter carries an inherent risk of colonization, which increases 5–10% per day of catheter duration [20]. Additionally, patients may experience lower urinary tract symptoms, bladder spasms, or catheter discomfort, which can mimic a UTI. Defining a true postoperative UTI in the setting of an existing urethral catheter can be difficult. Therefore, many urologists utilize prolonged AP postoperatively with the goal of decreasing the risk of postoperative infection. The AUA Best Practice Statement recognizes “anatomic anomalies of the urinary tract associated with risks of obstruction, poor drainage, or abnormal storage pressures” as well as preoperative colonization as risk factors for SSI.

Several retrospective studies have found SSI and UTI to be the most common postoperative complications after urethroplasty [4,5,6,21,22]. Rates of SSI and UTI have been reported at between 2.8 and 6.5% and between 3.4 and 6.9%, respectively. Several factors have been identified as predictive of increased risk of complications. In one study, preoperative bacteriuria was found to be an independent risk factor for postoperative infection (OR 1.81, *p* = 0.002) [4]. Calvo et al. (2024) specifically found that preoperative Gram-negative bacilli and *Enterococcus* were associated with increased risk for overall 90-day postoperative complications (14.2% risk) [23]. Additional risk factors include comorbid conditions (i.e., diabetes mellitus), operative time, and prior urethroplasty [4,24].

## 5. Antibiotic Prophylaxis After Urethroplasty: What Does the Evidence Show?

Table 2 summarizes the relevant literature regarding antibiotic prophylaxis after urethroplasty. The goal of AP post-urethroplasty is to reduce the risk of postoperative complications. Many urologists utilize prolonged AP until catheter removal. A prospective single-arm study by Kim et al. (2021) assessed the impact of a standardized AP protocol on infectious complications after urethroplasty. Their protocol included preoperative urine cultures and treatment for 3–5 days for any positive culture (24–48 h if only sensitive to IV antibiotics) [25]. Intraoperative prophylaxis included a cephalosporin (first, second, or third generation) or a fluoroquinolone for penicillin or cephalosporin allergies. Patients were discharged with a foley catheter on nitrofurantoin 100 mg BID until catheter removal, and ciprofloxacin or trimethoprim sulfamethoxazole was given on the day of catheter removal. They defined postoperative UTI as “>100 K CFU/mL of a single organism and at least one urinary symptom, which included suprapubic pain, flank pain, fever >101 F without other identified causes, dysuria that persists >2 days after catheter removal, or frequency or urgency that persists >2 days after catheter removal [25].” With this protocol, SSI and UTI rates were 4.1% and 6.7%, respectively [25]. Predictors of UTI in their multivariable model included preoperative UTI, coronary artery disease, and membranous urethroplasty. The most common organisms were *Escherichia coli*, *Staphylococcus aureus*, and *Klebsiella pneumoniae.*

Shorter-duration AP has not been associated with increased infectious complications. A study by Kim et al. (2022) compared continuous postoperative nitrofurantoin 100 mg BID until catheter removal in addition to ciprofloxacin or trimethoprim-sulfamethoxazole on the day of catheter removal to only ciprofloxacin or trimethoprim-sulfamethoxazole on the day of catheter removal [26]. They utilized the same definition of UTI as stated above. They found no significant difference in rates of UTI or SSI between AP regimens. Hanasaki et al. (2022) retrospectively reviewed 81 patients in whom postoperative AP was discontinued on postoperative day two and not resumed until the day before foley catheter removal. In their protocol, preoperative positive urine cultures were treated for 2–5 days; then, patients were treated with ampicillin or sulbactam perioperatively until postoperative day two. After discontinuation of antibiotics, they repeated a urine culture and treated prior to foley catheter removal either with (1) ampicillin or sulbactam if postoperative culture was negative or (2) with culture-directed antibiotics if the postoperative culture was positive. They found overall rates of SSI and UTI to be 3.7% and 2.5%, respectively, which is similar to prior studies with use of prolonged antibiotic regimens, despite only resuming antibiotics 1 day prior to catheter removal [27]. Baas et al. (2021) retrospectively compared patients who received extended AP until foley catheter removal versus those who received AP for 3 days surrounding catheter removal. Perioperatively, patients received one dose of cefazolin or ciprofloxacin. They found no difference in rates of UTI or wound infection between the two groups [28]. Of note, they treated postoperative UTI based on a positive culture or empirically for lower urinary tract symptoms, which may have overestimated the number of infections in the decreased AP group.

A feared complication of urethroplasty is stricture recurrence, which can occur in up to 25% of patients [24,29,30]. Postoperative UTI is thought to induce inflammatory changes at the urethral anastomosis, which has been purported to impair wound healing and increase risk of urethroplasty failure. A retrospective review of 398 patients undergoing urethroplasty by a single surgeon who provided AP until foley catheter removal found no association between stricture recurrence and postoperative positive urine culture or UTI over a median follow-up of 2.3 years [31]. Similarly, Galansky et al. (2024) retrospectively examined short- and long-term complications associated with AP in patients undergoing urethroplasty specifically with buccal mucosal graft (BMG) [32]. They found similar rates of UTI and SSI between groups and no difference in risk of recurrence or revision at 5 years. While mostly retrospective, the currently available literature shows no evidence that extended AP or postoperative UTI impacts stricture recurrence to date.

Providing AP is not benign. Harmful effects of extended AP include increased healthcare costs, potential for *Clostridium difficile* infection, and emergence of MDR organisms. Hoover et al. (2024) retrospectively compared patients post-urethroplasty who received 4 weeks of AP versus those who did not receive AP. They found no difference in postoperative complications including UTI, wound complications, and stricture recurrence [33]. They found a significantly higher number of MDR organisms in postoperative urine cultures for those who had received extended AP.

Overall, the existing evidence consisting of retrospective studies has shown no benefit of extended AP in preventing short-term infectious complications or long-term urethroplasty failure. Specifically, studies comparing shorter-duration AP to traditional extended AP found no difference in infectious complications. Furthermore, there is a real cost to extended AP regimens, as it does significantly increase antibacterial resistance, the emergence of MDR organisms, and the potential for opportunistic infections.

## 6. Antibiotic Prophylaxis in Other Urologic Procedures

Examining the evidence on antimicrobial prophylaxis in urologic procedures beyond urethroplasty significantly enhances the generalizability of findings across reconstructive urology. Many urologic interventions involve urinary tract reconstruction and necessitate prolonged catheterization, creating similar infection risk profiles despite different underlying pathologies. Currently, there is a striking lack of clear guidelines for these procedures, resulting in considerable variation in antibiotic prophylaxis practices among urologists. None of the studies reviewed, summarized in Table 3, have demonstrated clear benefit from prolonged prophylaxis across this spectrum of procedures—including vesicourethral reconstruction, bladder neck procedures, and hypospadias—which suggests a universal principle that may apply throughout urology.

### 6.1. Radical Prostatectomy

Post-radical prostatectomy (RP) patients routinely have foley catheters in place for 1–2 weeks to allow for healing of the vesicourethral anastomosis. There are no specific guidelines regarding AP at time of catheter removal after RP and there is wide variability amongst clinician practices, with most administering AP around the time of catheter removal. Several studies have explored different AP regimens post-RP, with conflicting results. One non-randomized study compared three days of ciprofloxacin to no AP and found that AP reduced symptomatic UTIs from 7.3% to 3.1% [34]. A randomized controlled trial by Berrando et al. (2019) comparing two doses of oral ciprofloxacin (one dose the evening before catheter removal and one dose the morning of catheter removal) versus no AP found no difference in the rate of symptomatic UTI between the control (5.95%) and AP groups (6.02%) within six weeks after catheter removal [35]. In this study, symptomatic UTI was defined as a positive culture with >100,000 colony forming units (CFU)/mL and at least one symptom or sign compatible with UTI (dysuria, frequency, urinary retention, fever, suprapubic pain, or hematuria). In a similar study, Ehdaie et al. (2022) cluster-randomized patients to receive 1-day versus 3-day regimens of oral ciprofloxacin surrounding catheter removal and examined rates of postoperative UTI. They found no difference in postoperative UTI between groups and therefore deemed the 1-day regimen non-inferior [36].

Similarly to the post-urethroplasty population, many post-RP patients have asymptomatic bacteriuria while the foley catheter is in place, even despite AP. In the randomized trial by Berrando et al. (2019), patients had urine cultures performed at time of foley removal, which were positive in 65% of patients in the control group and 29% of patients in the AP group, highlighting the common occurrence of asymptomatic bacteriuria [35]. Most of those who did go on to develop a symptomatic UTI had a different organism isolated at time of symptomatic culture. In a study by Banks et al. (2013), the researchers examined urine cultures at the time of foley catheter removal in patients post-RP. The patients were started on ciprofloxacin twice daily starting the night before catheter removal for a total of seven days [37]. A urine culture was collected immediately prior to foley catheter removal. Of 334 patients, 25% had positive cultures, of which 7% were resistant to ciprofloxacin. However, only two men had developed symptoms consistent with UTI prior to catheter removal [37]. Therefore, asymptomatic bacteriuria does not correlate with symptomatic UTI in these patients.

### 6.2. Hypospadias Repair

Research has shown that up to 90% of pediatric urologists utilize prolonged AP for patients undergoing hypospadias repair with postoperative stent or catheter placement [43]. Similarly to urethroplasty, AP is thought to prevent postoperative infectious complications and potentially reduce recurrence/stenosis. The evidence has been mixed regarding the utility of AP in preventing postoperative complications. Meir and Livne (2004) found a higher incidence of complicated UTI in patients who did not receive AP through catheter removal [38]. However, in a similar study, Kanaroglou et al. (2013) found no difference in rates of UTI or overall complications when comparing AP to no AP [39]. Zeiai et al. (2016) compared continuous AP versus a two-dose regimen at time of urethral stent removal and found no difference in UTI [40]. Roth et al. (2018) compared postoperative urine samples in patients who received trimethoprim-sulfamethoxazole versus those who did not after distal hypospadias with urethral stenting. They found a significantly higher rate of bacteriuria and pyuria in those who were not treated with AP; however, there were no differences in symptomatic UTI or wound complications between groups [41].

A double-blind, placebo-controlled, randomized trial was conducted on boys undergoing hypospadias repair with urethral stenting comparing trimethoprim-sulfamethoxazole to placebo for 10 days (duration of urethral stent placement) [42]. Outcomes included symptomatic UTI or SSI within 30 days postoperatively. Additional outcomes included urethrocutaneous fistula, meatal stenosis, urethral stricture, glans/urethral dehiscence, and/or urethral diverticulum. There were no significant differences between groups in postoperative UTI, SSI, or other complications [42]. Of note, the desired sample size was not reached due to inadequate recruitment; therefore, the trial did not have sufficient power.

Recently, Pogorelic et al. (2024) [44] compared the use of triclosan-coated polydioxanone (PDS Plus) versus uncoated polydioxanone (PDS II) sutures for prevention of SSI in hypospadias repair. Triclosan is an antiseptic substance that has been shown to reduce bacterial load and slow bacterial growth via inhibition of fatty acid synthesis. They found that patients who had undergone surgery using PDS Plus sutures had significantly lower SSIs compared to those in whom PDS II sutures were used [44]. This highlights the potential role of infection prevention adjuncts such as coated sutures and antibacterial washes in the reduction of SSI, which may also reduce the need for prolonged AP.

## 7. Future Directions

Future research should continue to elucidate meaningful post-surgical outcomes following urologic reconstruction and explore predictors associated with these clinically relevant metrics. Prospective, randomized trials are needed to better determine the need for AP, specifically in the context of prolonged foley catheter use and at time of removal. Subsequent investigative efforts should be directed toward establishing a pathogen-specific approach to antimicrobial prophylaxis and treatment across preoperative, intraoperative, and postoperative phases, thereby enhancing antimicrobial stewardship practices and mitigating the potential emergence of MDR organisms. High-quality research into this topic will lead to more detailed national and international guidelines on AP in these patients.

## 8. Conclusions

Patients undergoing urethroplasty are at risk for postoperative infectious complications given preoperative urinary stasis, baseline presence of urethral or suprapubic catheters, and postoperative need for prolonged catheter use. There are no guidelines regarding antibiotic prophylaxis post-urethroplasty and significant heterogeneity exists across urology practices. Research has not shown prolonged antibiotic prophylaxis to be clinically beneficial in reducing short-term infectious complications or long-term success. Antibiotic prophylaxis is not benign, given the risk of multidrug-resistant organisms and opportunistic infections. Reconstructive urologists should consider limiting antibiotic prophylaxis use after urethroplasty.

The growing body of evidence suggesting no clear benefit from extended antibiotic prophylaxis following urethral reconstruction suggests the need for a paradigm shift in clinical practice; any potential change needs to be guided by empirical evidence. Moving forward, our efforts should focus on rigorous prospective randomized trials comparing different prophylactic approaches while incorporating microbiome analysis to better understand the relationship between urinary bacterial flora and clinical outcomes. Such investigations may ultimately lead to more personalized, evidence-based antibiotic stewardship in reconstructive urology that minimizes unnecessary antimicrobial use while maintaining optimal patient outcomes. This shift toward targeted prophylaxis represents a crucial advancement in balancing infection prevention with antimicrobial stewardship principles.

## Figures and Tables

**Table 1:** Current societal recommendations regarding use of prophylactic antibiotics.

	Perioperative	Continuous During Postoperative Catheterization	At Time of Catheter Removal
**AUA** [3,9]	Single dose of a first- or second-generation cephalosporin	Not recommended	Consider in certain settings
**EAU** [14,15]	Perioperative antibiotics per local antibiotic resistance profile	Not recommended	Not specified
**IDSA** [16,17,18]	Single dose of a first-generation cephalosporin	Not recommended	Consider in certain settings

AUA = American Urological Association; EAU = European Association of urology; IDSA = Infectious Disease Society of America.

**Table 2:** Summary of studies assessing antibiotic prophylaxis for urethroplasty.

Study	Study Type	Preoperative Antibiotic Prophylaxis	Perioperative Antibiotic Prophylaxis	Postoperative Antibiotic Prophylaxis	Number of Patients	UTI Criteria	UTI Rate	Wound Infection Rate
Kim et al. (2021) [25]	Retrospective Review, Mult-institutional	UA/UCx within 3 weeks of surgery Preoperative UTI defined as >100 K CFU/mL regardless of symptoms without urinary catheter >50 K CFU/mL regardless of symptoms with a urinary catheter. Positive cultures treated for 3–5 days according to sensitivities, or 24–48 h if sensitive to IV antibiotics only	24 h of IV 1st-, 2nd-, or 3rd-generation cephalosporins (Fluoroquinolone if allergy)	Nitrofurantoin 100 mg BID until catheter removal (Keflex if allergic) 2 doses of Ciprofloxacin or Bactrim given on day of catheter removal	390	>100 K CFU/mL of a single organism + at least one urinary symptom (suprapubic pain, flank pain, fever >101 F, dysuria, frequency or urgency >2 days after catheter removal)	6.7%	4.1%
Kim et al. (2022) [26]	Retrospective Review, Multi-institutional	UA/UCx within 3 weeks of surgery Preoperative UTI defined as 3. >100 K CFU/mL regardless of symptoms without urinary catheter 4. >50 K CFU/mL regardless of symptoms with a urinary catheter. Positive cultures treated for 3–5 days according to sensitivities, or 24–48 h if sensitive to IV antibiotics only	24 h of IV 1st-, 2nd-, or 3rd-generation cephalosporins (Fluoroquinolone if allergy)	Cohort A: Nitrofurantoin 100 mg BID until catheter removal (Keflex if allergic) 2 doses of Ciprofloxacin or Bactrim given on day of catheter removal. Cohort B: 2 doses of Ciprofloxacin or Bactrim given on day of catheter removal	900	>100 K CFU/mL of a single organism + at least one urinary symptom (suprapubic pain, flank pain, fever > 101 F, dysuria, frequency or urgency >2 days after catheter removal)	Cohort A: 6.7% Cohort B: 3.9% (*p* > 0.05)	Cohort A: 4.1% Cohort B: 3.7% (*p* > 0.05)
Hanasaki et al. (2022) [27]	Retrospective, Single institution	UA/Ucx 1 month prior to surgery Positive cultures treated with IV antibiotics for 2–5 days	Ampicillin or sulbactam until postoperative day 2	Repeat urine culture after discontinuation of perioperative antibiotics If positive, culture-directed antibiotics beginning 1 day before catheter removal If negative, ampicillin or sulbactam ×3–4 days starting 1 day before catheter removal	81	Not defined	2.5%	3.7%
Baas et al. (2021) [28]	Retrospective, Single institution	Not defined	Cefazolin IV x 1 dose (ciprofloxacin for allergy)	Group 1: extended AP until catheter removal. Group 2: AP for 3 days around catheter removal (Bactrim twice daily, Keflex if allergic)	120	Positive culture or lower urinary tract symptoms treated empirically	Group 1: 6.7% Group 2: 11.7% (*p* > 0.05)	Group 1: 3.3% Group 2: 1.7% (*p* > 0.05)

AP = antimicrobial prophylaxis; CFU = colony forming units; IV = intravenous; UA = urinalysis; UCx = urine culture; UTI = urinary tract infection.

**Table 3:** Antibiotic prophylaxis in other urological procedures.

Study	Study Type	Preoperative Antibiotic Prophylaxis	Perioperative Antibiotic Prophylaxis	Postoperative Antibiotic Prophylaxis	Number of Patients	UTI Criteria	UTI Rate	Wound Infection Rate
Pinochet et al. (2010) [34]	Non-randomized comparative	Not specified	Intraoperative antibiotics (not specified)	3-day course of ciprofloxacin starting day before catheter removal (treatment group) vs. no antibiotics (control group)	713 (261 with ABT, 452 without)	Symptomatic UTI within 6 weeks after catheter removal	3.1% (ABT) vs. 7.3% (no ABT)	Not reported
Berrondo et al. (2019) [35]	Prospective randomized clinical trial	Not specified	Perioperative antibiotics (not specified)	2 doses of oral ciprofloxacin prior to urinary catheter removal (treatment group) vs. no antibiotics (control group)	175 (85 with ABT, 90 without)	Not clearly specified	No significant difference between groups	No significant difference between groups
Ehdaie et al. (2021) [36]	Cluster randomized trial	Not specified	Not specified	1-day vs. 3-day regimen of prophylactic antibiotics at catheter removal	Not specified in abstract	Positive urine cultures (≥10^5^ CFU) with at least 1 symptom: fever (>38 °C), urgency, frequency, dysuria or suprapubic tenderness	1-day not inferior to 3-day regimen	Not reported
Banks et al. (2013) [37]	Prospective observational	Not reported	Not reported	Prophylactic antibiotics 1 day before, day of, and 5 days after catheter removal	334	Not applicable (study focused on bacteriuria and resistance patterns)	Not the focus of study—examined bacteriuria rates; 25% had positive urine culture but only 0.6% developed UTI symptoms	Not reported
Meir & Livne (2004) [38]	Prospective RCT	None	Single-dose cefazolin	Group 1: None Group 2: 4 days oral cephalexin	89 patients Group 1: 52 Group 2: 37	Culture-proven	Group 1: 0% Group 2: 0%	Group 1: 0% Group 2: 0%
Kanaroglou et al. (2013) [39]	Retrospective Cohort	Not specified	Cefazolin	Group 1: None Group 2: TMP-SMX until catheter removal	407 patients Group 1: 213 Group 2: 194	Culture-proven with symptoms	Group 1: 3.3% Group 2: 2.6% (*p* = 0.66)	Group 1: 2.8% Group 2: 3.1% (*p* = 0.68)
Zeiai et al. (2016) [40]	Retrospective Cohort	Not specified	Not specified	Group 1: 5 days trimethoprim Group 2: 1-day trimethoprim	113 patients Group 1: 63 Group 2: 50	Not clearly defined	Group 1: 1.6% Group 2: 0%	Group 1: 3.2% Group 2: 6%
Roth et al. (2018) [41]	Prospective RCT	Not specified	Single dose cefazolin	Group 1: TMP-SMX for 9 days Group 2: No antibiotics	64 patients Group 1: 30 Group 2: 34	Positive culture with symptoms	Group 1: 0% Group 2: 2.9% (*p* = 1.0)	Group 1: 3.3% Group 2: 8.8% (*p* = 0.618)
Faasse et al. (2022) [42]	Multicenter Prospective RCT	Not specified	Cefazolin	Group 1: TMP-SMX for 10 days Group 2: Placebo for 10 days	166 patients Group 1: 81 Group 2: 85	Positive culture (>50,000 CFU/mL) with fever or symptoms	Group 1: 0% Group 2: 3.5% (*p* = 0.246)	Group 1: 2.5% Group 2: 7.1% (*p* = 0.28)

## Data Availability

No new data were created or analyzed in this study.

## Abbreviations

UTI	urinary tract infection
AP	antibiotic prophylaxis
AUA	American Urological Association
SSI	surgical site infection
MDR	multidrug-resistant
EAU	European Association of Urology
IDSA	Infectious Disease Society of America
CAUTI	catheter-associated urinary tract infection
IV	intravenous
BMG	buccal mucosal graft
RP	radical prostatectomy
CFU	colony forming units
PDS	polydioxanone
